# Human neural stem cells rapidly ameliorate symptomatic inflammation in early-stage ischemic-reperfusion cerebral injury

**DOI:** 10.1186/scrt519

**Published:** 2014-11-23

**Authors:** Lei Huang, Sunnie Wong, Evan Y Snyder, Milton H Hamblin, Jean-Pyo Lee

**Affiliations:** Tulane University School of Medicine, Center for Stem Cell Research and Regenerative Medicine; Pharmacology, 1430 Tulane Ave, SL-99, New Orleans, LA 70112 USA; Sanford-Burnham Medical Research Institute, Center for Neuroscience, Aging and Stem Cells, La Jolla, CA 92037 USA

## Abstract

**Introduction:**

Clinically, a good deal of injury from stroke results from ischemic-reperfusion. There is a loss of cerebral parenchyma and its associated cells, disruption of neuronal connections, compromise of the blood-brain barrier, and inflammation. We tested whether exogenously engrafted human neural stem cells could migrate rapidly and extensively to damaged regions, following transplantation into a neurogenic site where migration cues are already underway during stroke onset, then counteract a number of these pathological processes.

**Methods:**

One day post-injury, we injected human neural stem cells (hNSCs) into the ipsilesional hippocampus of a mouse model of stroke with middle cerebral artery occlusion to induce focal ischemia followed by reperfusion (MCAO/R). The time frame for hNSC transplantation corresponded to upregulation of endogenous proinflammatory cytokines. We examined the effect of hNSC transplantation on pathological processes and behavioral dysfunction 48 hours post-injury.

**Results:**

Twenty-four hours after transplantation, engrafted hNSCs had migrated extensively to the lesion, and infarct volume was reduced relative to MCAO/R controls. The behavioral deficits seen in MCAO/R controls were also significantly improved. Given this rapid response, we hypothesized that the mechanisms underlying therapeutic activity were anti-inflammatory rather than due to cell replacement. In support of this idea, in hNSC-transplanted mice we observed reduced microglial activation, decreased expression of proinflammatory factors (tumor necrosis factor-α, interleukin (IL)-6, IL-1β, monocyte chemotactic protein-1, macrophage inflammatory protein-1α) and adhesion molecules (intercellular adhesion molecule-1, vascular cell adhesion molecule-1), and amelioration of blood-brain barrier damage.

**Conclusions:**

While long-term effects of engrafted hNSCs on the amelioration of ischemic stroke-induced behavioral dysfunction in a rodent model have been reported, our study is the first to show rapid, beneficial impacts on behavioral function (within 24 hours) upon early delivery of hNSCs into the hippocampus.

**Electronic supplementary material:**

The online version of this article (doi:10.1186/scrt519) contains supplementary material, which is available to authorized users.

## Introduction

Neural stem cells (NSCs) possess multiple actions that are potentially therapeutic. These include functional neural replacement in multiple central nervous system (CNS) regions [1], as well as bystander effects. The bystander or ‘chaperone’ effects, previously reported by us and others, include delivery of therapeutic gene products inherently synthesized by stem cells, which might both directly protect endangered host cells and inhibit toxic components of the microenvironment (for example, anti-inflammatory actions) [2–6].

Stroke causes long-term neurological disability and is the second leading cause of mortality worldwide. Most strokes are ischemic and caused by thrombosis. Thrombolysis in occluded brain arteries can be an effective reperfusion treatment to salvage cells in the ischemic penumbra. However, fast reperfusion contributes to secondary injury by disrupting cerebral microvascular endothelial cell tight junctions that constitute the blood-brain barrier (BBB) accompanied by neuronal death [7]. Loss of BBB integrity promotes extravasation of fluids and intravascular proteins into the brain parenchyma. Compromise of the BBB in ischemic stroke may be mediated by multiple effectors, including growth factors, upregulation of endothelial-leukocyte adhesion molecules, inflammatory factors, matrix metalloproteinases, and disruption of tight junctions [8–13].

Reperfusion following ischemic injury causes biphasic opening of the BBB [14]. The initial stage of the transient and reversible BBB opening, which occurs several hours post-reperfusion, leads to an irreversible second phase of BBB opening following 24 hours to 72 hours post-reperfusion. The two openings of the BBB are separated by a refractory period. The second BBB opening contributes significantly to neural cell death. Thus, reducing inflammation during the initial stage and ameliorating any BBB opening may lessen further neuronal damage.

NSC transplantation offers a novel, therapeutic strategy for early-stage ischemic stroke, when inflammatory signals are prominent, by dampening the inflammatory response. To be therapeutically effective during this time, engrafted NSCs must migrate quickly and extensively into the lesioned area. Others have reported on their efforts to engraft exogenous NSCs post-ischemia into various regions of the brain in stroke models [15, 16]. Yet, reported outcomes repeatedly show an insufficient number of stem cells migrating into stroke lesions and poor recovery of behavioral functions 24 hours post-transplantation. None of these reports describe NSCs being engrafted into the hippocampal region of the brain as we report here.

In this study, we investigated the role of human NSCs (hNSCs) on the expression of adhesion molecules, proinflammatory cytokines and BBB repair in a mouse model of ischemic/reperfusion cerebral injury. Animal models of transient focal ischemia induced by middle cerebral artery occlusion with subsequent reperfusion (MCAO/R) are well-defined and widely used [17]. Our study was designed to determine the effects of hNSCs on multiple secondary pathological processes 24 hours to 48 hours post-MCAO/R, the time frame that coincides with the second phase of BBB opening. No studies to date have shown extensive dissemination of intracranially injected hNSCs within 24 hours post-stroke. Here, we used well-characterized hNSCs of fetal origin. We transplanted hNSCs into the ipsilesional hippocampus after a 60-minute MCAO/24-hour reperfusion. We evaluated how rapidly and efficiently transplanted hNSCs migrated into the stroke lesion and determined their pathophysiology as it relates to BBB disruption, neuroinflammation and behavioral dysfunction.

## Materials and methods

### Animals

Male C57BL/6J mice, 24 to 26 g, (Jackson Laboratory, Bar Harbor, ME, USA) were maintained at 20 ± 2°C and on a 12 hour light-dark cycle with food and water *ad libitum*.

### Animal model of stroke

All experimental protocols were approved by the Institutional Animal Care and Use Committee of Tulane University (New Orleans, LA, USA). All experiments and animal handling were performed in accordance with the guidelines of the Tulane University Protocol, American Veterinary Medical Association and National Institutes of Health Guide for the Care and Use of Laboratory Animals.

Focal cerebral ischemia was induced by intraluminal MCAO, as described previously [18]. Briefly, mice were anesthetized with 1% isoflurane in 30% oxygen using a face mask. The left common carotid artery and external carotid artery were exposed through a midline neck incision. A 6-0 nylon monofilament coated with silicon rubber (Doccol Corporation, Sharon, MA, USA) was introduced into the left internal carotid artery through the external carotid stump to occlude the origin of the MCA. After a 60-minute occlusion, reperfusion was introduced by filament withdrawal. Sham-operated control mice underwent a similar surgery but the filament was removed immediately after insertion (no occlusion, no reperfusion). Body temperature was continuously maintained at 37 ± 0.5°C. Regional cerebral blood flow (rCBF) through the MCA was assessed by laser Doppler (Perimed, Stockholm, Sweden). Successful MCAOs resulted in an over 80% decrease in rCBF after MCA occlusion and a post-MCAO/R recovery to 90.1% ± 4.0. Brains were collected 48 hours post-MCAO/R for quantitative analyses.

### Human neural stem cell culture

We used well-characterized hNSCs of fetal origin (generated from fetal CNS by the Snyder Lab). Specifically, primary hNSCs were dissociated from the telencephalon of late first-trimester human fetal cadavers as previously described [2], particularly the ventricular zone, which is rich in NSCs. The hNSCs were expanded and maintained in defined medium: (D)MEM/F12 high glucose (Invitrogen, Carlsbad, CA, USA), recombinant human epidermal growth factor (EGF) (20 ng/mL, Invitrogen), recombinant basic fibroblast growth factor (bFGF) (20 ng/mL, Invitrogen), leukemia inhibitory factor (LIF; 10 ng/ml, Millipore, Billerica, MA, USA), heparin (8 μg/ml, Sigma, St Louis, MO, USA), L-glutamine (Invitrogen), and antibiotics (penicillin, streptomycin, and amphotericin C). Prior to confluence, cells were transferred enzymatically once per week during refeeding or within 48 to 72 hours prior to transplantation. RT-PCR and immunostaining confirmed nestin, Sox2, glial fibrillary acidic protein (GFAP), doublecortin, EGF receptor and nucleostemin expression during the expansion phase. Flow cytometry showed high levels of neural stem cell markers (for example, CD133). These cells have not been genetically manipulated, carry no transgenes and are propagated with mitogens alone. The ability of hNSCs to engraft successfully (xenograft) and integrate in the mouse brain has been previously documented [2, 19]. Their genetic fingerprints and karyotypes have remained stable over many years. No non-neural cell types or tumors were seen following engraftment.

### Human neural stem cell transplantation

We transplanted hNSCs 24 hours post-MCAO/R into the hippocampus, a neurogenic site where signals for stem cell migration are already underway. Adult C57BL/6J mice were anesthetized with isoflurane and placed in a stereotaxic frame (Stoelting Co., Wood Dale, IL, USA). Ocular ointment was applied to the eyes to prevent drying, animal heads were wiped with 70% ethanol, and the skin on the head was cut open at the midline. A small burr hole (0.5 mm diameter, F.S.T.) was drilled into the skull (2 mm posterior to bregma; 1.5 mm lateral to sagittal suture). A total of 2 μl of PBS containing approximately 100,000 viable cells were injected over a 3-minute period into the ipsilesional hemisphere of the MCAO/R-treated brain (depth; 2 to 2.5 mm dorsal). Mock transplantation controls received the same volume of PBS. The wound was closed with cyanoacrylate glue (Vector Laboratories, Burlingame, CA, USA), and brains were collected 48 hours post-MCAO/R for analysis.

### Behavioral tests

We performed adhesive removal, beam walk, and rotarod behavioral tests on each mouse for 3 days consecutively before MCAO (as training) and from 2 to 31 days post-injury. For the adhesive removal test, a small piece (3 mm × 4 mm) of adhesive tape was applied using equal pressure to the contralateral forepaw [20]. The mouse was then placed in a transparent Perspex box to record the time for the mouse to remove the tape (to a 120-second maximum). For the beam walk test, mice were trained to traverse an elevated beam (700-mm long and 5-mm wide) to reach an enclosed box. Following training, mice that reached the box in 20 seconds without stopping were selected for the study. After MCAO/R, latency to traverse the beam (to a 60-second maximum) was recorded. The rotarod test [21] was performed with some modifications: four velocities (10, 15, 20, and 25 rpm) were used, and three trials were performed per velocity. The time a mouse remained on the rod was recorded; the trial ended if the mouse fell from the rod. Speed averages are presented as a percentage relative to the sham mice. Data are presented as total amount of time on the rod as a percentage relative to the sham mice.

### Tissue processing

At 48 hours post-MCAO/R (24 hours post-transplantation), mice were deeply anesthetized and transcardially perfused, first with normal saline, then with phosphate-buffered 4% paraformaldehyde (PFA). Brains were post-fixed in 4% PFA for 24 hours and cryoprotected in 30% sucrose; free-floating coronal sections (30 μm) were prepared using a vibratome (Leica VT1000s, Heidelberg, Germany); frozen sections were embedded in optimum cutting temperature (O.C.T.) compound (Tissue-Tek, Thermo Fisher Scientific, USA) and cut coronally at a 20-μm thickness.

### Quantification of infarct volume

Infarct volume was measured by cresyl violet staining [22]. Mice were sacrificed 48 hours post-MCAO/R by transcardial perfusion, brains postfixed in 4% PFA for 24 hours, and 30-μm coronal vibratome sections cut at five levels (Bregma 1.78, 0.98, 0.14, -1.22, and -1.94 mm). In addition, triphenyl tetrazolium chloride (TTC) staining was performed. Coronal sections of 1 mm were cut 48 hours post-MCAO/R and incubated in 2% TTC solution (Sigma). In viable brain tissue, TTC is converted by mitochondria to a red substance, while the ischemic area remains colorless. Infarct size was measured using the National Institutes of Health (NIH) program ImageJ. Infarct volume was calculated as a percent volume of the contralateral hemisphere to compensate for edema, using the following formula: [(volume of contralateral hemisphere - (volume of total ipsilesional hemisphere – volume of infarct area)]/volume of contralateral hemisphere.

### Immunohistochemistry

Nonspecific binding to 30-μm coronal free-floating sections was blocked by incubation in 10% goat serum in PBS, pH 7.4, containing 0.1% Tween 20, 0.3% Triton X-100. Sections were then incubated with primary antibodies at 4°C overnight. Primary antibodies used (and dilutions) were rabbit anti-Iba-1 (1:300, Wako, Osaka, Japan), mouse anti-human cytoplasm Stem121 (1:500, Stem Cells, Inc., Palo Alto, CA, USA), rabbit anti-human mitochondria (1:300, Millipore), and rabbit anti-BDNF (1:400, Abcam, Cambridge, MA, USA). Sections were then incubated in biotinylated goat anti-rabbit immunoglobulin G (IgG) antibody (1:600, Vector Laboratories), followed by avidin-peroxidase (ABC, Vector Laboratories). The reaction products were visualized with 3, 3-diaminobenzidine (DAB, Sigma). Immunofluorescent staining included the secondary antibodies goat anti-rabbit IgG Alexa Fluor 594 (1:500, Invitrogen) and goat anti-mouse IgG Alexa Fluor 488 (1:500, Invitrogen).

### Reverse transcriptase polymerase chain reaction

Total RNA was extracted from ipsilesional MCAO/R mouse brain tissue. Brain tissue (approximately 20 to 30 mg) preserved in RNAlater (Ambion, Inc., Austin, TX, USA) was immersed in Trizol reagent (Invitrogen) and homogenized using MagNA Lyser (Roche Diagnostics, Indianapolis, IN, USA). After chloroform extraction, RNA fractions were mixed with an appropriate volume of 70% ethanol and loaded onto an RNeasy column (Qiagen, Valencia, CA, USA). The retained material was digested with DNase I (Qiagen). To prepare first-strand cDNA, 2 μg of total RNA was reverse-transcribed with ABI high-capacity cDNA reverse transcriptase and random primers (4368814, Invitrogen). PCR amplification was performed with 2 μl of reverse transcriptase reaction mixture in 10 μl of SsoFast Probes Supermix with Rox (Biorad, Hercules, CA, USA) in a CFX96 Real-time thermal cycler (Biorad). PCR reaction conditions included a 30-second hold at 95°C; 35 cycles of activation for 5 seconds at 95°C and annealing/extending for 10 seconds at 60°C. TaqMan^®^ Gene Expression Assays (Applied Biosystems, Foster City, CA, USA) were used (TNF-α: Mm00443258_m1; IL-6: Mm00446190_m1; IL-1β: Mm00434228_m1; VCAM-1: Mm01320970_m1; ICAM-1: Mm00516023_m1; Ccl2 (MCP1): Mm00441242_m1; Ccl3 (MIP-1α): Mm00441259_g1; GAPDH: Mm99999915_g1). Ct values were normalized relative to GAPDH. The Livak (2^-ΔΔCt^) method was used to calculate changes in target gene expression relative to sham mice.

### Blood-brain barrier permeability assay

BBB integrity was assessed by IgG immunostaining with minor modifications [23] and Texas Red^®^ (Invitrogen, Carlsbad, CA, USA) dextran perfusion [24]. Free-floating sections were incubated with biotinylated horse anti-mouse IgG (BA-2000, 1:500, Vector Laboratories) at 4°C overnight following blocking in 10% normal horse serum and 1% BSA in PBS, pH 7.4. After rinsing in 0.3% H_2_O_2_ for 30 minutes to quench endogenous peroxidase activity, sections were incubated with avidin-biotin horseradish peroxidase complex (ABC, Vector Laboratories) for 30 minutes, then visualized with DAB. Using ImageJ, the extent of BBB damage was quantified as the percentage of area of IgG positive staining per ipsilateral hemisphere. For Texas Red dextran perfusion, mice were deeply anesthetized with isoflurane, and Texas Red dextran 70 kDa (D1864, Invitrogen) in PBS (50 mg/ml) was injected into the inferior vena cava for 2 minutes of circulation. Mice were immediately killed by decapitation. Brains were quickly extracted and post-fixed in 4% PFA for 24 hours then cryoprotected by 30% sucrose for 48 hours. After O.C.T. embedding, brains were cut coronally in 20-μm sections. Slices were directly coverslipped using mounting media with 4′,6-diamidino-2-phenylindole (DAPI) (H-1200, Vector Laboratories).

### Western blot analysis

At 48 hours post-MCAO/R, mice were deeply anesthetized and the ipsilesional cortex and hippocampal regions were collected (Bregma: -1.5 mm to 3.5 mm). Total protein was isolated by homogenization in cold NP-40 lysis buffer (Boston Bioproducts Inc., Boston, MA, USA). Supernatants were collected after centrifugation at 12,000 rpm for 15 minutes. A total of 30 μg protein was loaded onto 4% to 12% Bis-Tris NuPAGE Novex gels (Invitrogen) and transferred onto NC membranes (Invitrogen). Primary antibodies used include the following: MMP-9 (1:1000, Abcam), Iba-1 (1:500, Abcam), CD11b (1:1000, Abcam), ZO-1 (1:500, Invitrogen), BDNF (1:1000, Abcam), and β-actin (1:2500, Thermo Fisher Scientific, Carlsbad, CA, USA). (Invitrogen, Carlsbad, CA, USA) Blots were probed with appropriate horseradish peroxidase-conjugated secondary antibodies (1:3000, Invitrogen) and detected using the ECL Western blot substrate (Thermo Fisher Scientific). Protein band intensities were quantified using ImageJ.

### Gelatin zymography

Functional matrix metalloproteinase-9 (MMP-9) enzyme was detected via SDS-PAGE zymography, as described previously [25]. Brain samples were prepared for Western blotting, except that supernatants were not denatured prior to gel loading. Protein (30 μg/well) was loaded onto a Novex 10% gelatin zymogram gel (Invitrogen). After electrophoresis, the gel was renatured with renaturing buffer (Invitrogen) and incubated in developing buffer (Invitrogen) at 37°C overnight. The gel was stained with 0.5% SimplyBlue SafeStain (Invitrogen) for 60 minutes, then destained. MMP-9 protease activity was visualized indirectly as clear bands against a dark background. Images were analyzed using ImageJ.

### Confocal microscopy

Images were captured with a Bio-Rad MRC 1024 equipped with a single photon Kr/Ar laser and a Bio-Rad Radiance 2100MP equipped with a multiphoton laser. Images were acquired sequentially to negate channel cross-talk. Pixel resolution was 1024 × 1024, with negative and positive control images taken at the same settings. Each merged image was visualized using orthogonal projections composed of 9 to 16 optical Z-planes of 0.5 to 1 μm thickness.

### Statistical analysis

Statistical analyses were performed using GraphPad Prism 6 and SPSS 19 software. One-way analysis of variance (ANOVA) with Fisher’s least significant difference (LSD) *post-hoc* test assessed differences between multiple groups. Tests were considered statistically significant at *P*-values <0.05. Data are presented as mean ± SEM.

## Results

### Transplanted human neural stem cells ameliorate neurological dysfunction in a stroke model

**Figure 1:**
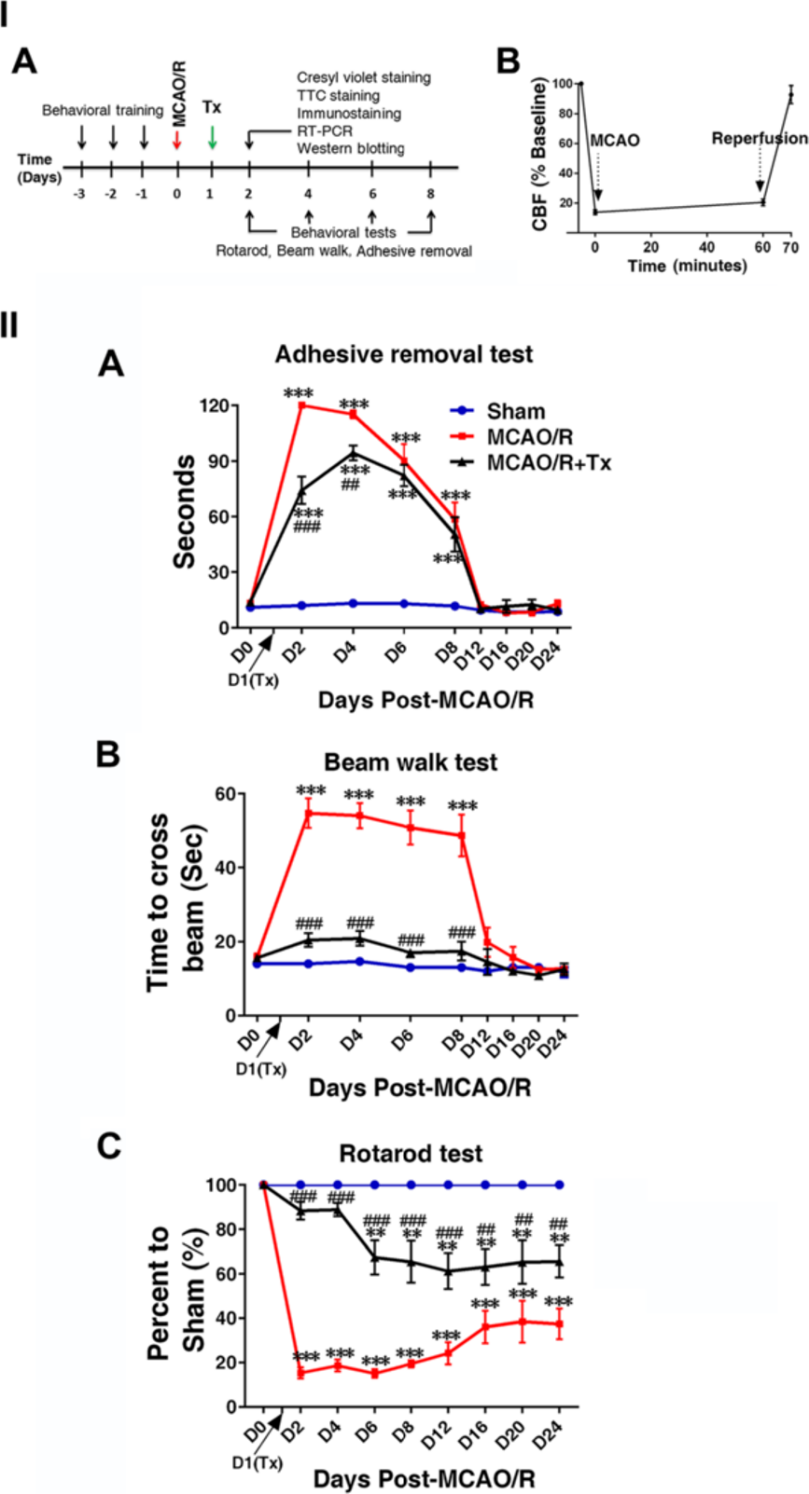
**Human NSC transplantation ameliorates behavioral deficits. I. (A)** Experimental timeline. MCAO/R was performed in C57BL/6 J mice at time 0, and hNSCs were transplanted into the ipsilesional hippocampus 24 hours later. Outcomes were assessed at the indicated intervals. Tx, transplantation. **(B)** Relative cerebral blood flow (rCBF) was measured by laser Doppler flowmetry over the area supplied by the MCA. Pre-ischemic rCBF was assigned a value of 100%. Subsequent values are presented as a percentage of that value. MCAO promoted an 80% decrease in rCBF (86.1 ± 1.6%), and the reperfusion flow rate 60 minutes post-MCAO was 90.1 ± 4.0% (n = 18). **II.** **(A)** In the forepaw adhesive removal tests, the mean adhesive removal time for hNSC-transplanted mice was significantly shorter than that of MCAO/R mice, showing improvement (n = 14). **(B)** In the beam walk tests, hNSC-transplanted MCAO/R mice improved neurological dysfunction as shown by crossing the beam in significantly less time than did nontransplanted MCAO/R mice (n = 14). **(C)** In the rotarod tests, hNSC-transplanted mice improved motor dysfunction as shown by remaining on the rod for a significantly longer time than did nontransplanted MCAO/R mice (n = 14). Four velocities (10, 15, 20, and 25 rpm) were used, and three trials were performed at each velocity. Durations at all speeds are summed, and data are presented as a percentage relative to the sham mice. **P <0.01, ***P <0.001 versus sham mice; ^##^ *P* <0.01; ^###^ *P* <0.001 versus nontransplanted MCAO/R mice. Data are expressed as mean ± SEM. MCA, middle cerebral artery; MCAO/R, middle cerebral artery occlusion with reperfusion; NSC, neural stem cells; SEM, standard error of the mean.

Ischemic-reperfusion damage in rodents correlates with neurological dysfunction [26, 27]. We first investigated hNSC effects on behavioral dysfunction caused by stroke. We performed behavioral tests of sensorimotor, motor and balance beginning one day after hNSC transplantation into the hippocampus (Figure 1 IA and IB). We pinpointed the hippocampus, a naturally supportive niche of NSCs, as the ideal injection site because signals for stem cell proliferation and migration are widespread there.

Each mouse was subjected to three behavioral tests for three consecutive days before surgery (pretraining) and from 2 to 31 days post-surgery. The effects of transplanted hNSCs for improving neurological dysfunction were clearly evident on days 2 to 8 post-injury, and efficacy of transplanted hNSCs on behavioral dysfunction was maintained beyond 12 days. Thus, here we focused on assessing the impact of hNSCs at early time points post-stroke (Figure 1 II). Results for hNSC-transplanted MCAO/R mice showed amelioration of behavioral impairment.

For the forepaw adhesive removal test, which assesses sensorimotor deficits, mice were subjected to MCAO/R with and without hNSC transplant. The mean time to remove adhesive tape for MCAO/R mice was greater compared with sham mice (*P* <0.001 at days 2, 4, 6, and 8; Figure 1 IIA). However, mean removal time was significantly less for hNSC-transplanted (MCAO/R + Tx) mice compared to nontransplanted MCAO/R mice (*P* <0.001 at day 2; *P* <0.01 at day 4; Figure 1 IIA), demonstrating improved behavioral function following hNSC transplantation.

The beam walk test assesses balance by measuring the time required for an animal to walk across an elevated narrow beam to a platform [28]. Mean walk time in MCAO/R mice significantly increased compared with sham controls (*P* <0.001). However, mean walk time significantly decreased in hNSC-transplanted mice on days 2 to 8 compared with nontransplanted MCAO/R mice, demonstrating improved performance following hNSC transplantation (*P* <0.001; Figure 1 IIB).

The rotarod test evaluates balance and motor coordination by forcing mice to remain on a rotating rod. The time duration on the rod dramatically decreased in nontransplanted MCAO/R mice compared with sham mice starting at 2 days post-injury and remained low for the entire test period (*P* <0.001; Figure 1 IIC). In contrast, hNSC-transplanted (MCAO/R + Tx) mice showed improved performance, although not recovering to sham control levels, as shown by these mice, which remained on the rod significantly longer than did MCAO/R mice (*P* <0.01 at day 24; Figure 1 IIC). Sham mice showed no behavioral dysfunction. The improved behavioral function observed with hNSC transplantation persisted beyond 1 month.

Thus, the present study suggests that transplanting hNSCs into the hippocampus at 24 hours post-MCAO/R can provide long-term benefits. We used heat-killed hNSCs as a negative control to show that effects demonstrated by engrafted hNSCs in this study are not due to inflammatory responses induced by cell transplantation. When dead hNSCs were transplanted, there was no improvement in behavioral dysfunction (data not shown). Furthermore, in order to validate that human NSC xenotransplantation is not suboptimal in the mouse brain, we compared the effect of hNSCs with that of mouse NSCs (mNSCs). We transplanted mNSCs, C17.2, which are well-characterized and widely used [2, 29], and obtained similar results showing that stroke mice engrafted with mNSCs performed significantly better than nontransplanted MCAO/R mice. Based on these findings, we used hNSCs for this study.

### Human neural stem cells engraft widely following ischemia and are associated with reduced infarct volume

**Figure 2:**
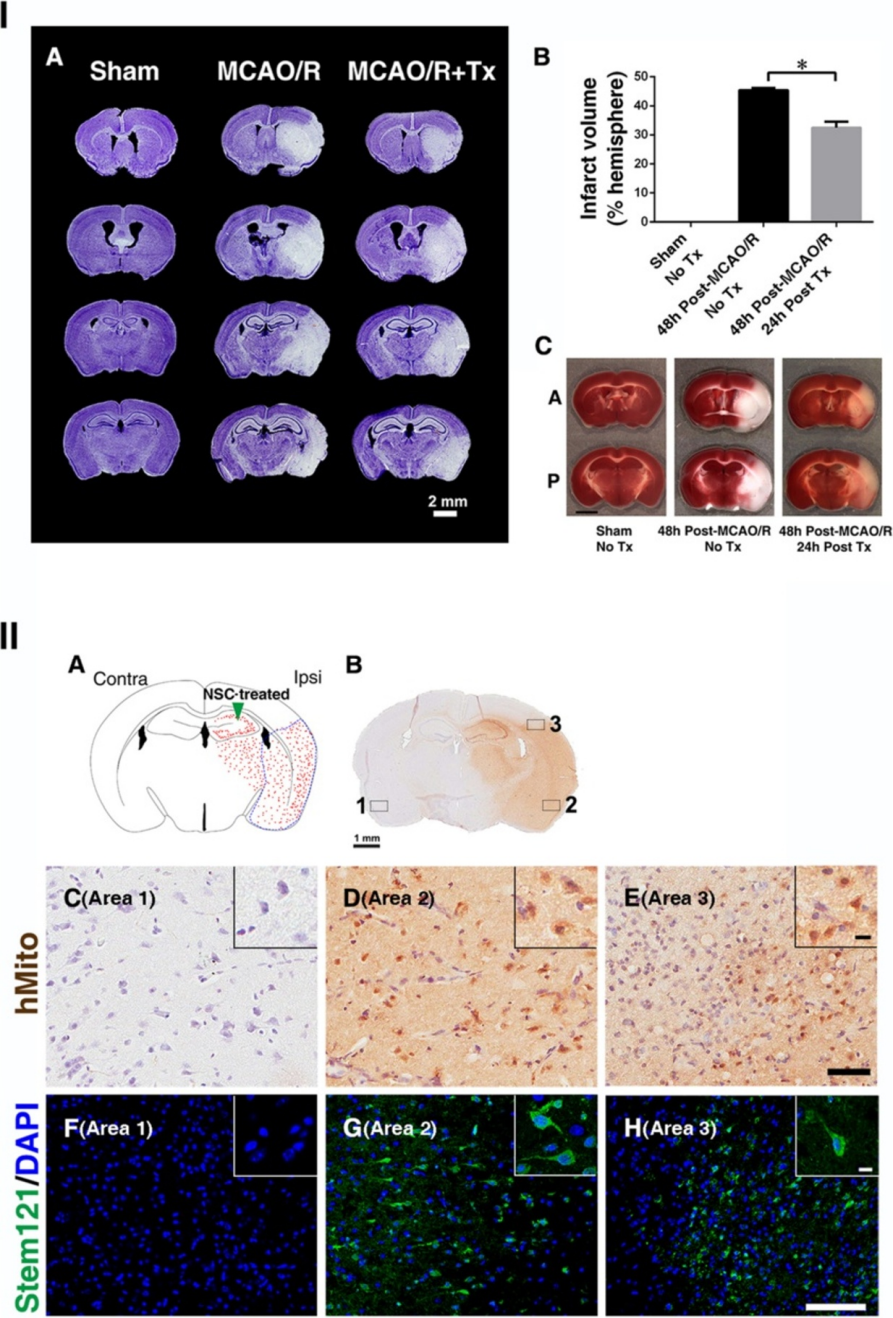
**Human NSCs engraft widely and reduce infarct volume.** **I.** **(A)** Infarct volume was reduced in hNSC-transplanted mice (MCAO/R + Tx) 24 hours post-transplantation (neurons are stained with cresyl violet, purple; infarct, white). **(B)** Quantification of II A. (n = 4, *P <0.05). **(C)** TTC staining (white, infarct). Shown are two representative samples of the same brain from anterior to posterior. A, anterior; P, posterior. **II. (A)** Diagram of brain and injection site. Arrowhead indicates NSC injection site; red dots, hNSC-disseminated areas. Ipsi, ipsilesional; Contra, contralesional. **(B-H)** hNSCs were identified with two different human-specific antibodies (human mitochondria, hMito or human cytoplasmic protein, Stem121). Three sampling sites are shown in rectangles, **(C-H)** higher magnification images: area 1, (no infarct), area 2 (infarct center), area 3 (peri-infarct). **(B-E)** Immunoreactivity to human mitochondria (hMito; DAB, brown) and **(F-H)** to Stem121 (G, H: green). Scale bars: **B**, 1 mm; **E**, 50 μm (10 μm inset); **H,** 100 μm (10 μm inset). DAB, 3,3-diaminobenzidine; MCAO/R, middle cerebral artery occlusion with reperfusion; NSCs, neural stem cells; TTC, triphenyl tetrazolium chloride; Tx: Transplantation.

We next explored potential mechanisms underlying this behavioral improvement. We investigated the effects of hNSCs on infarct volume 24 hours post-transplantation (48 hours post-MCAO/R). We measured infarct volumes by staining with cresyl violet (white, infarct) (Figure 2 IA). Similar to previously reported results, 60 minutes of intraluminal MCAO induced ischemic lesions in both the caudo-putamen and cortex but not in the hippocampus [30]. Compared to nontransplanted MCAO/R mice, infarct size in transplanted brains was significantly reduced. The mean infarct volume (white areas) in MCAO/R mice was 45.38 ± 0.7% in the ipsilesional hemisphere and 32.5 ± 2.1% in hNSC-transplanted mice (*P* <0.05; Figure 2 IB). Cresyl violet staining patterns agreed with TTC staining. In viable brain tissue, TTC is converted by mitochondria to a red substance, but remains colorless in the ischemic area (Figure 2 IC; Additional file 1: Figure S1).

We next examined the distribution of engrafted hNSCs by immunostaining with two different human-specific antibodies (human mitochondria, hMito, or the human cytoplasmic protein, Stem121). We found that transplanted hNSCs (24 hours post-MCAO/R into the hippocampus) migrated throughout the ischemic lesions (Figure 2 IIB-H). It is anticipated that the earlier the intervention to prevent BBB damage, the better the clinical outcome post-stroke. However, regarding injection time points (other than at 24 hours post-MCAO/R), hNSCs migrated less extensively to the injured site when injected 6 hours or 12 hours post-MCAO/R than at 24 hours (analyzed 24 hours post-transplantation) [see Additional file 2: Figure S2]. Regarding injection sites other than the hippocampus, when hNSCs were transplanted into the ipsilesional striatum or lateral ventricle 24 hours post-MCAO/R, there was less extensive migration to the injured sites compared with hNSC injection into the hippocampal region [see Additional file 3: Figure S3].

### Human neural stem cell transplantation reduces levels of proinflammatory cytokines

**Figure 3:**
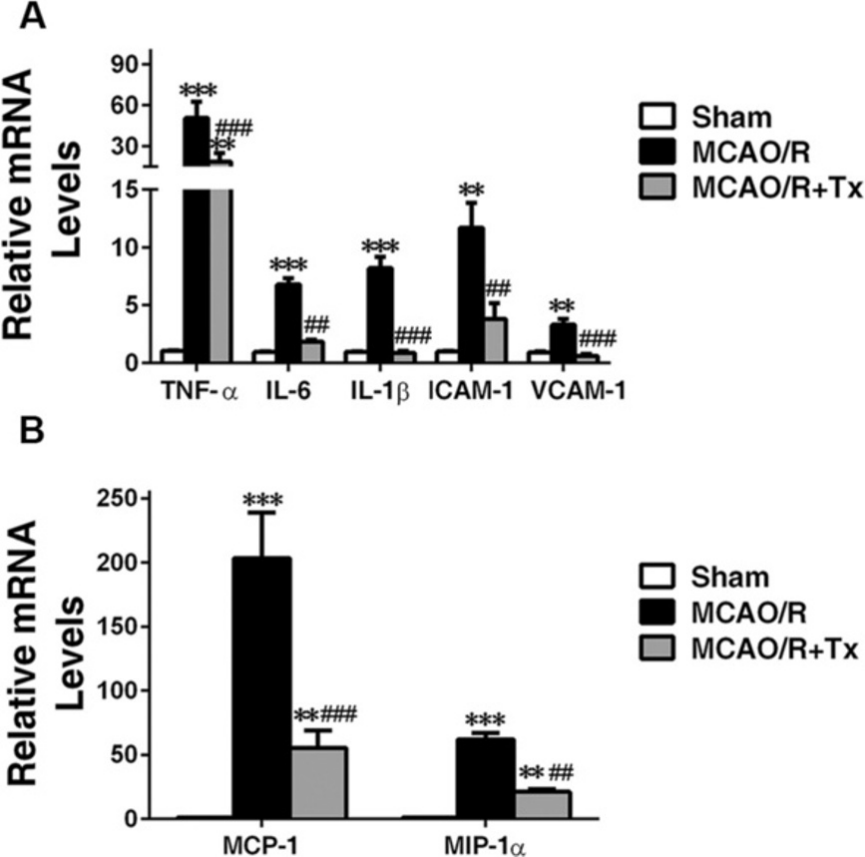
**Human NSC transplantation reduces proinflammatory gene expression. (A)** Reduced inflammatory marker expression in the ipsilesional hemisphere of hNSC-transplanted MCAO/R brains. Expression of inflammatory genes was measured by RT-PCR and normalized to that of GAPDH. Transcript levels of proinflammatory cytokines TNF-α, IL-6, IL-1β, and cell adhesion molecules, ICAM-1 and VCAM-1, are elevated in MCAO/R brains and downregulated in MCAO/R + hNSC-transplanted brains compared with MCAO/R brains. **P <0.01, ***P <0.001 versus sham mice; ^##^ *P* <0.01, ^###^ *P* <0.001 versus MCAO/R mice. **(B)** Chemokines MCP-1 and MIP-1α are dramatically elevated in the ipsilesional hemisphere 48 hours post-MCAO/R and significantly downregulated in hNSC-transplanted brains. **P <0.01, ***P <0.001 versus sham mice; ^##^ *P* <0.01, ^###^ *P* <0.001 versus MCAO/R mice. All data are expressed as mean ± SEM (n = 5). MCAO/R, middle cerebral artery occlusion with reperfusion; NSC, neural stem cells; SEM, standard error of the mean; Tx, transplantation.

Upregulation of proinflammatory cytokines and chemokines is elicited by ischemia and reperfusion [8–11, 13]. We examined the effect of hNSC transplantation on inflammation using RT-PCR. Forty-eight hours post-MCAO/R and without hNSC transplantation, expression of proinflammatory cytokines (TNF-α, IL-6, and IL-1β) was upregulated in the ipsilesional hemisphere by 50.8 ± 11.77, 6.8 ± 0.6, and 8.2 ± 0.6 fold, respectively, compared to sham animals (*P* <0.001; Figure 3A). By contrast, hNSC transplantation reduced expression of these genes (*P* <0.01; Figure 3A). Compared to sham animals, MCAO/R resulted in an upregulation of expression of the cell adhesion molecules, ICAM-1 and VCAM-1, by 11.7 ± 1.7 and 3.3 ± 0.3 fold, respectively (*P* <0.01). However, compared to MCAO/R mice, hNSC transplantation reduced expression of these genes by 7.9 ± 2.0 and 2.7 ± 0.3 fold, respectively (*P* <0.01; Figure 3A). Levels of mRNAs encoding chemokines MCP-1 and MIP-1α, indicators of microglial/macrophage activation, also significantly increased in MCAO/R mice by 203.3 ± 27.5 and 61.8 ± 3.3 fold, compared with sham mice (*P* <0.001; Figure 3B). hNSC transplantation significantly downregulated expression of both chemokines (*P* <0.01; Figure 3B). These findings indicate that transplanting hNSCs following injury decreases gene expression associated with inflammation and microglial/macrophage activation.

### Human neural stem cell transplantation ameliorates BBB damage

**Figure 4:**
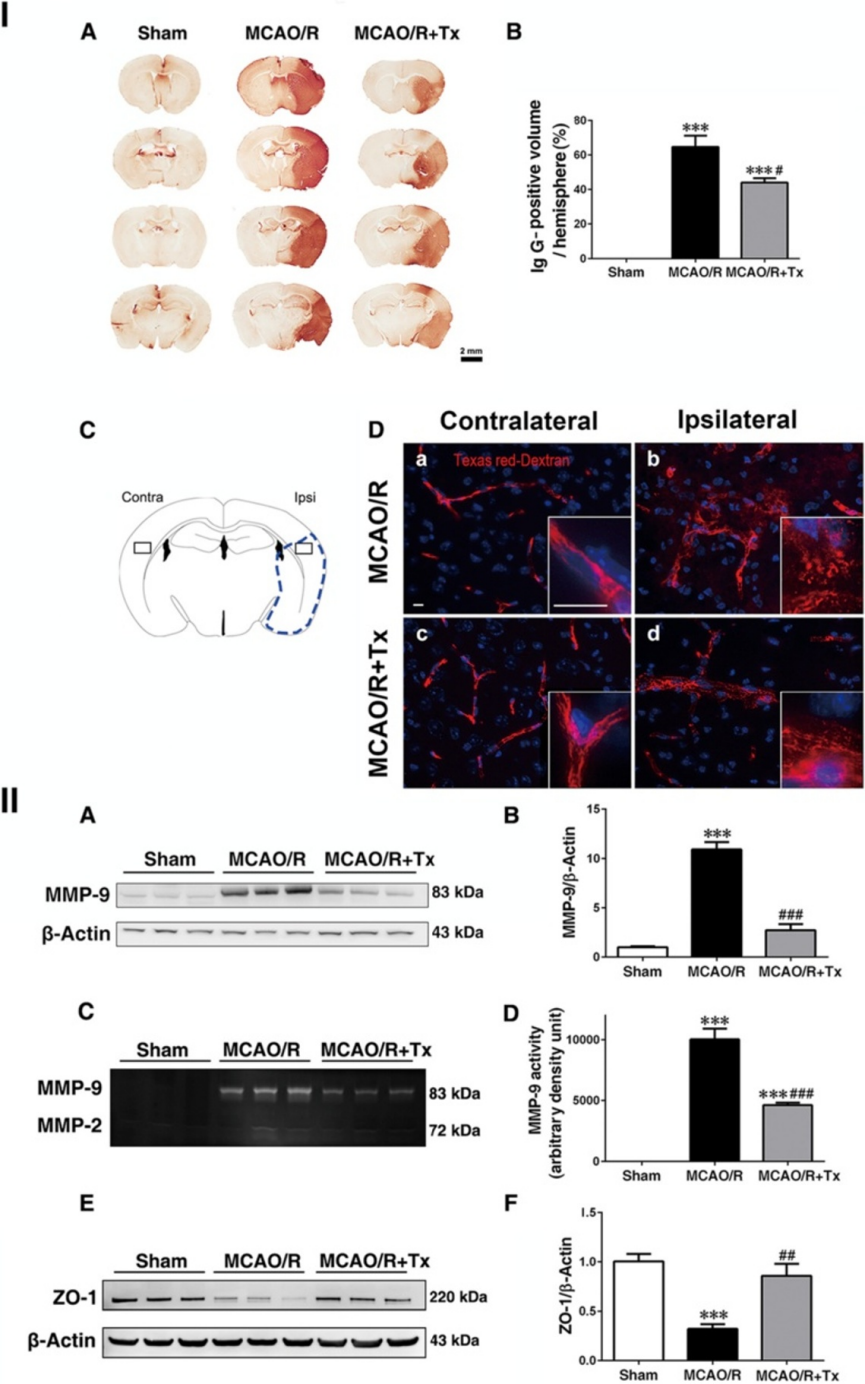
**Human NSC transplantation ameliorates BBB leakage. I. (A)** Representative images of IgG staining in sham, MCAO/R and MCAO/R + hNSC-transplanted (Tx) mice showing BBB damage. Scale bar, 2 mm. **(B)** Quantification of IgG staining, expressed as a percentage of the volume showing IgG-positive staining per ipsilesional hemisphere (n = 5, ***P <0.001 versus sham; ^#^ *P* <0.05 versus MCAO/R). **(C)** Brain diagram. **(D)** The spatial distribution of intravenously administered Texas Red dextran 70 kDa (red) in contralateral (**a, c**) and ipsilateral (**b, d**) cortical regions of stroke mice with/without hNSC transplantation 48 hours post-MCAO/R. Reduced extravasation was observed in hNSC-transplanted mice. Scale bar, 10 μm (10 μm, inset). **II. (A)** Western blot analysis showing MMP-9 protein level in the ipsilesional cortex. **(B)** Quantification of **A** (n = 6, ***P <0.001 versus sham; ^###^ *P <*0.001 versus MCAO/R). **(C)** Zymography assay showing MMP-9 activity in the ipsilesional cortex. **(D)** Quantification of **C** (n = 6, ***P <0.001 versus sham; ^###^ *P* <0.001 versus MCAO/R). **(E)** Western blot analysis of ZO-1. **(F)** Quantification of **E** (n = 4, ***P <0.001 versus sham; ^##^ *P* <0.01 versus MCAO/R). BBB, blood-brain barrier; IgG, immunoglobulin G; MCAO/R, middle cerebral artery occlusion with reperfusion; MMP-9, matrix metalloproteinase-9; NSC, neural stem cells; ZO-1, zona occluden.

Proinflammatory cytokines and chemokines that are upregulated following ischemic injury increase BBB permeability [31, 32]. To determine the effect of hNSCs on BBB permeability, we evaluated the extravasation of the blood-borne substance IgG into the brain parenchyma. Forty-eight hours post-MCAO/R, IgG increased in the ipsilesional hemisphere, as indicated by increased IgG immunostaining (Figure 4 IA), in MCAO/R brains compared to sham controls. Transplanted hNSCs reduced IgG levels (Figure 4 IA). The percentage of the IgG-positive area within the ipsilesional hemisphere was 64.73 ± 6.51% in MCAO/R brains (*P* <0.001) but decreased to 44.01 ± 2.52% in hNSC-transplanted brains (*P* <0.05; Figure 4 IB). BBB function was also assessed with intravenous injection of the fluorescent tracer Texas Red dextran (70 kDa). In the contralateral cortex, tracer was confined to the lumen of capillaries and showed intact vascular morphology (Figure 4 ID, a and c). MCAO/R caused massive tracer diffusion out of the leaky capillaries, which are shown as blurred and punctuated signals alongside the vascular segments in the ipsilateral cortex (Figure 4 ID, b). However, transplantation of hNSCs clearly reduced this diffusion, indicating improved BBB function (Figure 4 ID, d).

Following stroke, MMPs are upregulated in ischemic regions where they degrade the extracellular matrix, which plays an important role in maintaining BBB integrity. Thus, inhibiting MMPs reduces infarct size [33]. We determined the effect of hNSCs on MMP-9 and MMP-2, proteins that participate in BBB breakdown following ischemic stroke. Western blot analysis showed that MMP-9 levels in the MCAO/R group were increased 48 hours post-injury, compared to the sham group (*P* <0.001; Figure 4 IIA and B). Yet transplanted hNSCs significantly downregulated MMP-9 levels (*P* <0.001; Figure 4 IIA and B). To validate this finding, we used gel zymography (gelatin as a substrate) to measure MMP-9 enzymatic activity and found higher activity at 48 hours post-MCAO/R, compared to sham controls (*P* <0.001; Figure 4 IIC and D); transplanted hNSCs significantly decreased MMP-9 activity in MCAO/R mice (*P* <0.001, Figure 4 IIC and D). As to MMP-2 expression and activity, it was low at 48 hours post-MCAO/R (consistent with previous studies by others), suggesting that at this time point MMP-2 does not play a major role in the process (Figure 4 IIC).

Tight junctions strengthen the cerebral microvascular endothelial cell wall that defines the BBB [34]. BBB leakage *in vivo* without disrupting claudin-5 and occludin networks was reported 24 hours post-MCAO [24]. However, the tight junction protein zona occluden (ZO-1), which links the actin cytoskeleton and transmembrane protein occludin [35], is reportedly degraded by MMP-9 [36]. We investigated the effect of hNSCs on ZO-1 using Western blot analysis and found decreased ZO-1 levels in MCAO/R mice (Figure 4 IIE and F); however, transplanted hNSCs prevented ZO-1 degradation (*P* <0.01, Figure 4 IIE and F). Our Western blot analyses showed no significant differences in the levels of claudin-5 and occludin between groups (data not shown), suggesting that positive hNSC effects on these protein complexes relate to maintenance of their connection to the actin cytoskeleton.

**Figure 5:**
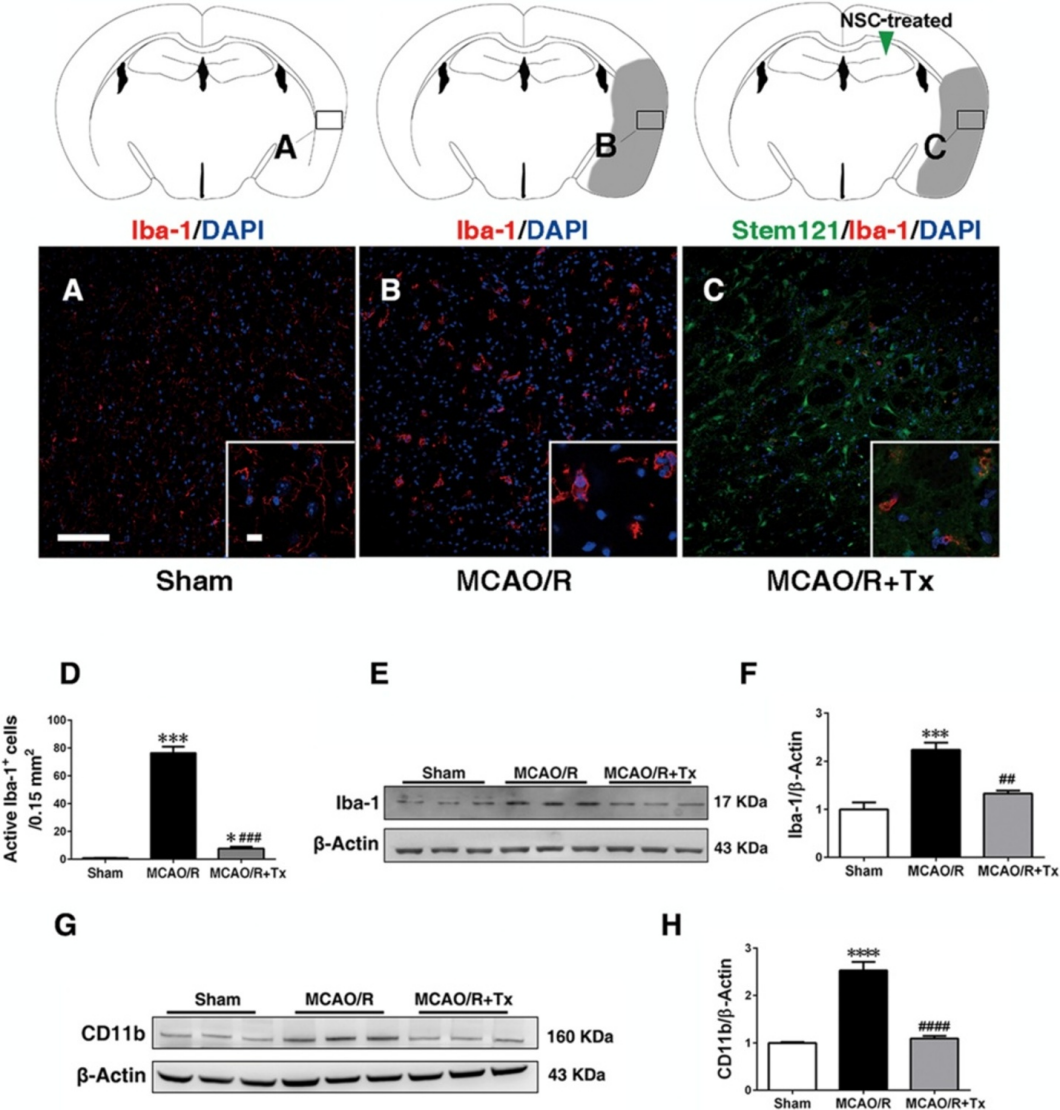
**Human NSC transplantation reduces microglial activation.** Immunofluorescence staining for Iba-1 (red), a microglial marker, and Stem121, an hNSC marker, **(A)** sham, **(B)** MCAO/R, and **(C)** MCAO/R + hNSC engrafted mice (Insets, higher magnification). Microglial activation was increased 48 hours post-MCAO/R, while hNSC transplantation suppressed activation by 24 hours post-transplantation (arrowhead, hNSC transplantation site). **(D)** Quantification of Iba-1-positive active microglia per area (0.15 mm^2^) in different mouse groups (n = 4, *P <0.05, ***P <0.001 versus sham; ^###^ *P* <0.001 versus MCAO/R). **(E)** Western blot analysis showing the Iba-1 protein level in the ipsilesional cortex. **(F)** Quantification of **E** (n = 3, ***, *P* <0.001 versus sham group; ##, *P* <0.01 versus MCAO/R group. **(G)** Western blot analysis showing the CD11b protein level in the ipsilesional cortex. **(H)** Quantification of **G** (n = 3, ****, *P* <0.0001 versus sham group; ####, *P* <0.0001 versus MCAO/R group). Data expressed as mean ± SEM. Rectangles indicate sampling regions. Gray region indicates infarct. MCAO/R, middle cerebral artery occlusion with reperfusion; NSC, neural stem cell; SEM, standard error of the mean.

We then asked whether transplanted hNSCs could reduce the number of activated inflammatory cells. Immunostaining of sham brains with the microglial/macrophage marker, Iba-1 indicated the presence of resting microglia (ramified shape) (Figure 5A). In contrast, following MCAO/R, the number of activated inflammatory cells (Iba-1-positive and amoeboid shape) dramatically increased throughout the infarct, compared with the sham mice (*P* <0.05, Figure 5B and D). Additionally, transplanted hNSCs significantly reduced the number of Iba-1-positive activated cells (*P* <0.001; Figure 5C and D). Consistent with this finding, Western blot analysis showed reduced Iba-1 and CD11b expression (another activated microglia/macrophage marker), indicating reduced inflammation in hNSC-engrafted brains (*P <*0.01, Figure 5E-H).

**Figure 6:**
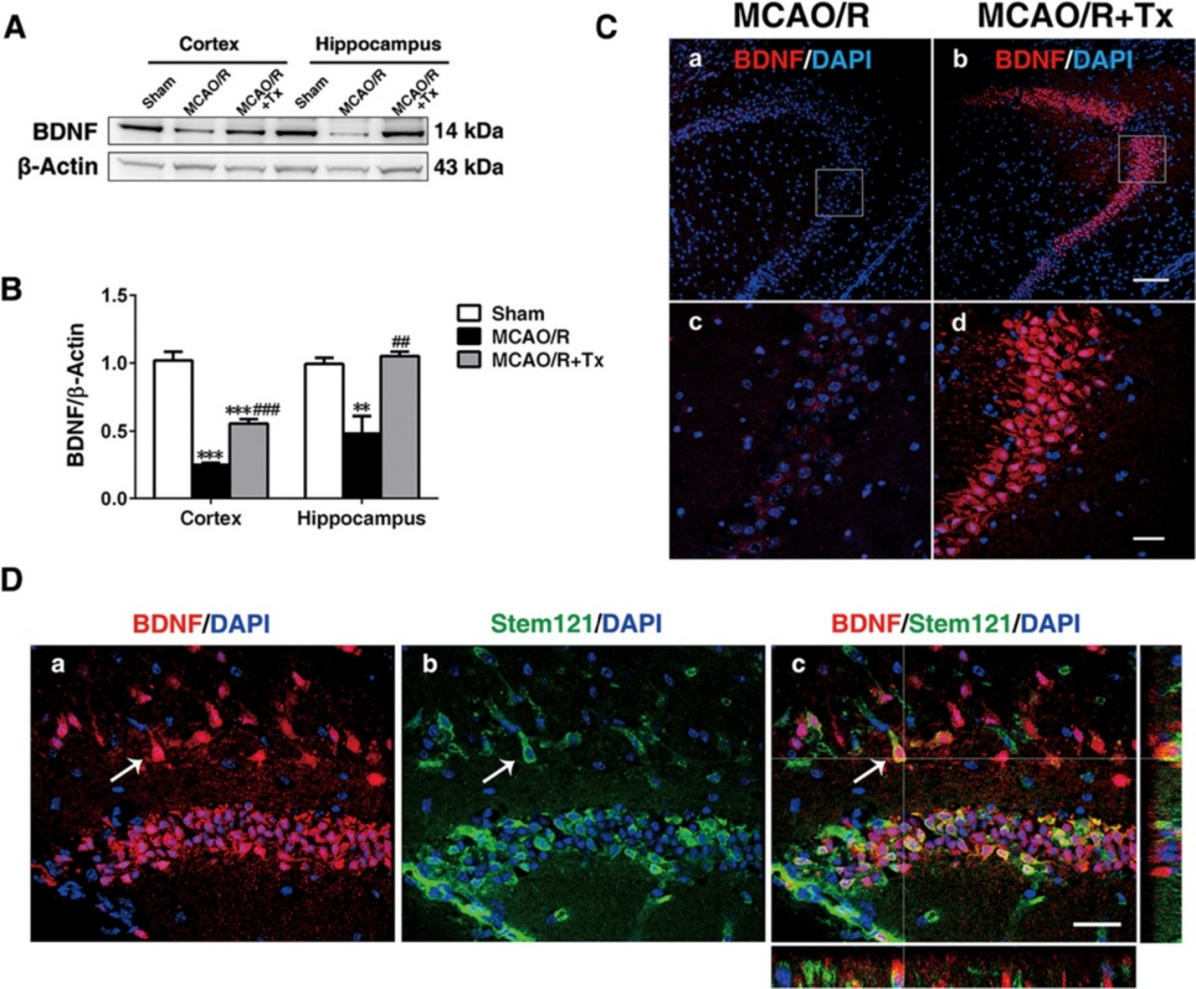
**Human NSC transplantation increases BNDF expression in the ipsilesional hemisphere. (A)** Representative Western blot showing BDNF expression in the cortex and hippocampus. **(B)** Quantification of **A**. (n = 4; **P <0.01, ***P <0.001 versus sham; ^##^ *P* <0.01, ^###^ *P* <0.001 versus MCAO/R). **(C)** Representative confocal photomicrographs showing BDNF (red) immunofluorescence in the ipsilesional hippocampus. DAPI staining is shown in blue (**c, d**: High magnification of rectangles shown in **a**, **b**, respectively). Scale bar, 100 μm (**a**, **b**); 30 μm (**c, d**). **(D)** BDNF-positive endogenous cells (red), Stem121-positive hNSCs (green), and BDNF-positive hNSCs, as indicated by arrow (arrow, yellow; merge). Scale bar, 30 μm. BNDF, brain-derived neurotrophic factor; DAPI, 4′,6-diamidino-2-phenylindole; MCAO/R, middle cerebral artery occlusion with reperfusion; NSC, neural stem cell.

Brain-derived neurotrophic factor (BDNF) is a major neurotrophin and promotes functional recovery and neuroprotection after stroke [37]. Thus, we analyzed and found reduced BDNF levels in MCAO/R brains 2 days post-injury (Figure 6). Transplanted hNSCs increased BDNF protein levels in the ipsilesional cortex and hippocampus compared to equivalent regions in MCAO/R brains (*P* <0.001 in cortex; *P* <0.01 in hippocampus, Figure 6A and B).

## Discussion

Most neurovascular diseases are complex and engage multiple processes leading to symptoms, making hNSCs promising for stroke therapy. The rodent MCAO model is typically used for focal cerebral ischemia studies [17], although infarct progression is faster than in other species. We explored the effects of hNSC intracranial (hippocampal) transplantation following MCAO with subsequent reperfusion (MCAO/R) in mice and found that hNSCs transplanted 24 hours post-stroke extensively and rapidly migrated to injured sites, intermingled with host cells, and lessened pathophysiological symptoms 48 hours post-MCAO/R. Consequently, transplanted stroke mice rapidly recovered sensorimotor deficits.

The acute phase of transient cerebral ischemia/reperfusion involves an inflammatory response accompanying BBB damage and leukocyte infiltration from the periphery [38]. Initial BBB leakage concurs with brain cell death; 4 hours after reperfusion coincides with dextran extravasation post-stroke in mice [24]. These studies indicate that to prevent ischemic brain cell death, NSC therapeutic action must take place within 4 hours of reperfusion. While neuroinflammatory signals may recruit transplanted NSCs to the infarct area after cerebral ischemia [19], hNSCs transplanted within 12 hours post-MCAO have limited migration. Therefore, we transplanted fetal hNSCs 24 hours post-MCAO/R and found rapid and extensive cell migration into the stroke lesion.

Regarding transplantation location, we found that the strategic area for hNSC injection is in the ipsilesional hippocampus. Others have engrafted exogenous NSCs into the brain post-ischemia [15, 16], but these transplantations were not performed in the hippocampus. Resulting outcomes repeatedly showed few NSCs migrating into the stroke lesion, thus preventing fast recovery of behavioral function. We, too, found poor hNSC migration when hNSCs were transplanted into various locations (for example, ventricle, striatum, cortex, or infarct core). We also claim the hippocampus as the ideal site for this type of intervention because it is one of only two regions in the adult mammalian brain where neurogenesis is ongoing even under normal circumstances. Further, it is abundant in signals for migration, proliferation, differentiation, and integration. In contrast, the subventricular zone (SVZ), which is the other area of ongoing adult neurogenesis, harbors only those neural precursors (at least in the rodent) that travel solely to the olfactory bulb. Other precursors there become only glia. Furthermore, it is debatable whether the human SVZ can give rise to neurons, whereas it is well-established that the human hippocampus will do so. Thus, the hippocampus has the potential to treat human conditions and provide the gateway for effective clinical intervention.

We previously reported on the anti-inflammatory role of engrafted hNSCs in CNS disorders [2–4]. Here, we further demonstrated that transplanted hNSCs ameliorate microvascular inflammation and BBB damage. Proinflammatory cytokines overproduced in the ischemic brain alter endothelial cell-matrix interactions and mediate neuronal cell death [8, 9]. We showed that hNSC engraftment downregulated expression of proinflammatory cytokines (TNF-α, IL-6, IL-1β). Endothelial-leukocyte adhesion molecules (ICAM-1 and VCAM-1) mediate leukocyte rolling, adhesion and transendothelial migration of leukocytes [10]. These adhesion molecules are elevated post-stroke. We now have found that transplanted hNSCs reduce expression of these factors.

Monocyte chemoattractant protein-1 (MCP-1, also known as CCL2) can recruit monocyte lineage cells following an inflammatory stimulus and contribute to ischemic stroke injury [11]. Macrophage inflammatory protein-1 α (MIP-1 α) has a neutrophil chemokinetic function [13]. Thus, downregulation of MCP-1 and MIP-1α following stroke would likely reduce transit of neutrophils and monocytes into the brain. Compatible with these findings, we observed a decreased number of Iba-1-positive activated myeloid cells following hNSC transplantation, compared with nontransplanted MCAO/R brains.

MMPs, a family of zinc-binding proteolytic enzymes, are implicated in ischemic injury. Elevated MMP-9 level and activity correlates with BBB breakdown after stroke [12, 36]. We showed that engrafted hNSCs significantly reduced the stroke-induced elevated MMP-9 level 48 hours post-MCAO/R, suggesting that hNSCs can ameliorate BBB disruption by inhibiting the elevated enzyme’s associated harmful events. MMP-9 induction is linked with tissue plasminogen activator (tPA)-induced hemorrhage in stroke patients [39] and animal models [40, 41]. Thus, our studies showing an MMP-9 reduction suggest that hNSC treatment could be a potential adjunct therapy following tPA treatment in the acute stage of ischemic stroke.

The tight junctions formed by cerebral microvascular endothelial cells define the BBB [34], and the proteins claudin-5 and occludin are integral to tight junction integrity. MMP-9 mediates BBB damage by degrading tight junctions [42]. BBB leakage occurs 24 hours post-MCAO despite the absence of a disrupted claudin-5 network and any reduction in occludin [24]. However, MCAO/R does degrade the tight junction protein ZO-1, which connects the actin cytoskeleton and transmembrane protein occludin [35]. MMP-9 reportedly degrades ZO-1 [36], suggesting a link between MMP-9 expression in stroke and BBB leakage. In support of this idea, we found increased ZO-1 expression 48 hours post-MCAO/R in hNSC-transplanted brains, signifying a potential role for hNSCs in protecting ZO-1 from degradation. Consistent with previous findings, our Western blot results showed no significant differences in claudin 5 or occludin levels. Furthermore, recovery of BBB integrity was confirmed by the much reduced extravasation of both IgG and fluorescent dextran (injected) in hNSC-engrafted brains 48 hours post-MCAO/R. Our results suggest that engrafted hNSCs can ameliorate secondary inflammatory brain damage by reducing cytokine production and ameliorating later phase BBB leakage. Furthermore, neurotrophins promote functional recovery after stroke [37]. As well, we observed significantly increased BDNF expression 24 hours post-transplantation, suggesting that BDNF upregulation may add a protective mechanism to this system.

Certainly, improving the physiological parameters for transplanting hNSCs following MCAO/R is important but improved behavior is the ultimate readout of any stroke therapy. We found that neurological function rapidly improved in mice 48 hours post-MCAO/R after hNSC transplantation at 24 hours, compared with nontransplanted MCAO/R controls. Although survival of engrafted stem cells was reduced between 3 to 5 days post-transplantation, behavioral function recovery persisted during the 1 month we monitored. This suggests that early intervention (as reported here) is paramount for lasting positive outcomes in stroke patients.

Current therapy using recombinant tPA has a less than 5 hour therapeutic window [43]. However, potential side effects of tPA include exacerbated BBB damage through leakage [44]. Our data show reduced pathophysiology and behavioral deficits seen 24 hours after hNSC transplantation (48 hours post-MCAO/R), suggesting that the therapeutic window for hNSC-mediated protection is much longer. Thus, the multifaceted actions attributed to hNSCs could offer a novel adjunct therapy after tPA treatment alone or if the window for tPA is missed.

There is a growing recognition in the stem cell field that NSCs (and other stem cell types) have multiple actions beyond simply neuronal replacement in the CNS. The teleological role of NSCs is not only to produce neurons but to produce support cells that elaborate homeostasis-promoting factors for their clonally-related progeny (for example, trophic factors, neuroprotective factors, and anti-inflammatory factors) [2, 4–6]. In some cases, as demonstrated here, those actions can be even more impactful than neuronal replacement.

## Conclusions

We have shown that transplanting hNSCs into the hippocampus 24 hours post-ischemia resulted in both fast and far-reaching migration to sites of pathology and a lessening of pathophysiological symptoms. hNSCs made quick work of reducing inflammation in ischemic-reperfusion cerebral injury via downregulating proinflammatory cytokines and ameliorating BBB damage. This important finding demonstrates not only the role of the NSC but its intrinsic biological action. Here we have been able to harness that capacity for a very important clinical problem: reperfusion injury after stroke.

## Electronic supplementary material

Additional file 1: Figure S1: Human NSC transplantation reduces infarct volume. Infarct volume in MCAO/R mouse brains was measured using TTC staining (Red, viable tissue; White, infarct). Sixty minutes of MCAO and 48-hour reperfusion induced ischemic damage in both the caudo-putamen and cortex. Infarct volume was reduced in hNSC-transplanted mice (MCAO/R + Tx) 24 hours post-transplantation. Sections are from the same animal and are shown from anterior to posterior (top to bottom). A, anterior, P, posterior. Shown are **(A)** sham, **(B, C)** MCAO/R, and **(D**, **E)** MCAO/R with transplantation. Tx: transplantation, Scale bar, 3 mm. (TIF 3 MB)

Additional file 2: Figure S2: hNSC transplantation 24 hours post-MCAO/R shows the most extensive migration into stroke lesion. **(A-C)** Human NSCs were identified with the human cytoplasmic marker, Stem121 (green), following different transplantation time points: **(A)** 6 hours, **(B)** 12 hours, **(C)** 24 hours post-MCAO/R. (Insets, higher magnification images). White arrow indicates Stem121-positive cells. Sampling sites are shown in rectangle of diagram. Ipsi, ipsilesional; Contra, contralesional. Scale bar, 10 μm (100 μm inset). (TIFF 1 MB)

Additional file 3: Figure S3: hNSCs transplanted into hippocampus 24 hours post-MCAO/R migrate most extensively into stroke lesion. **(A-C)** Diagrams show the distribution of NSCs 24 hours post-transplantation into the ipsilateral hippocampus, striatum, and lateral ventricle, respectively. Green arrows indicate transplantation site. Red dots indicate hNSC-disseminated areas. Ipsi, ipsilesional; Contra, contralesional. **(D-F)** hNSCs were identified with the human cytoplasmic marker, Stem121 (green). White arrows indicate Stem121-positive cells. The sampling sites are shown in the inset rectangles of A-C. Insets show higher magnification images. MCAO/R, middle cerebral artery occlusion with subsequent reperfusion. Scale bar, 10 μm (100 μm inset). Tx, transplantation. (TIF 1 MB)

## Abbreviations

BBB: blood-brain barrier
BDNF: brain-derived neurotrophic factor
BSA: bovine serum albumin
CCL2: chemokine ligand 2
CNS: central nervous system
DAB: 3,3-diaminobenzidine
(D)MEM: (Dulbecco’s) modified Eagle’s medium
EGF: epidermal growth factor
FGF: fibroblast growth factor
GAPDH: glyceraldehyde 3-phosphate dehydrogenase
GFAP: glial fibrillary acidic protein
hNSCs: human neural stem cells
Iba-1: ionized calcium-binding adapter molecule-1
ICAM-1: intercellular adhesion molecule-1
IL-1β: interleukin-1 beta
IL-6: interleukin-6
LIF: leukemia inhibitory factor
MCAO/R: middle cerebral artery occlusion/reperfusion
MCP-1: monocyte chemotactic protein-1
MIP-1α: macrophage inflammatory protein-1 alpha
MMP: matrix metalloproteinase
mNSCs: mouse neural stem cells
PBS: phosphate-buffered saline
PFA: paraformaldehyde
rCBF: regional cerebral blood flow
RT-PCR: reverse transcription polymerase chain reaction
SVZ: subventricular zone
TNF-α: tumor necrosis factor-alpha
tPA: tissue plasminogen activator
TTC: triphenyl tetrazolium chloride
VCAM-1: vascular cell adhesion molecule-1.
