# Supplementary figures and images for: Human neural stem cells rapidly ameliorate symptomatic inflammation in early-stage ischemic-reperfusion cerebral injury
Source: Stem Cell Res Ther. 2014 Nov 23;5(6):129. doi: 10.1186/scrt519 (PMC4445985; doi:10.1186/scrt519)

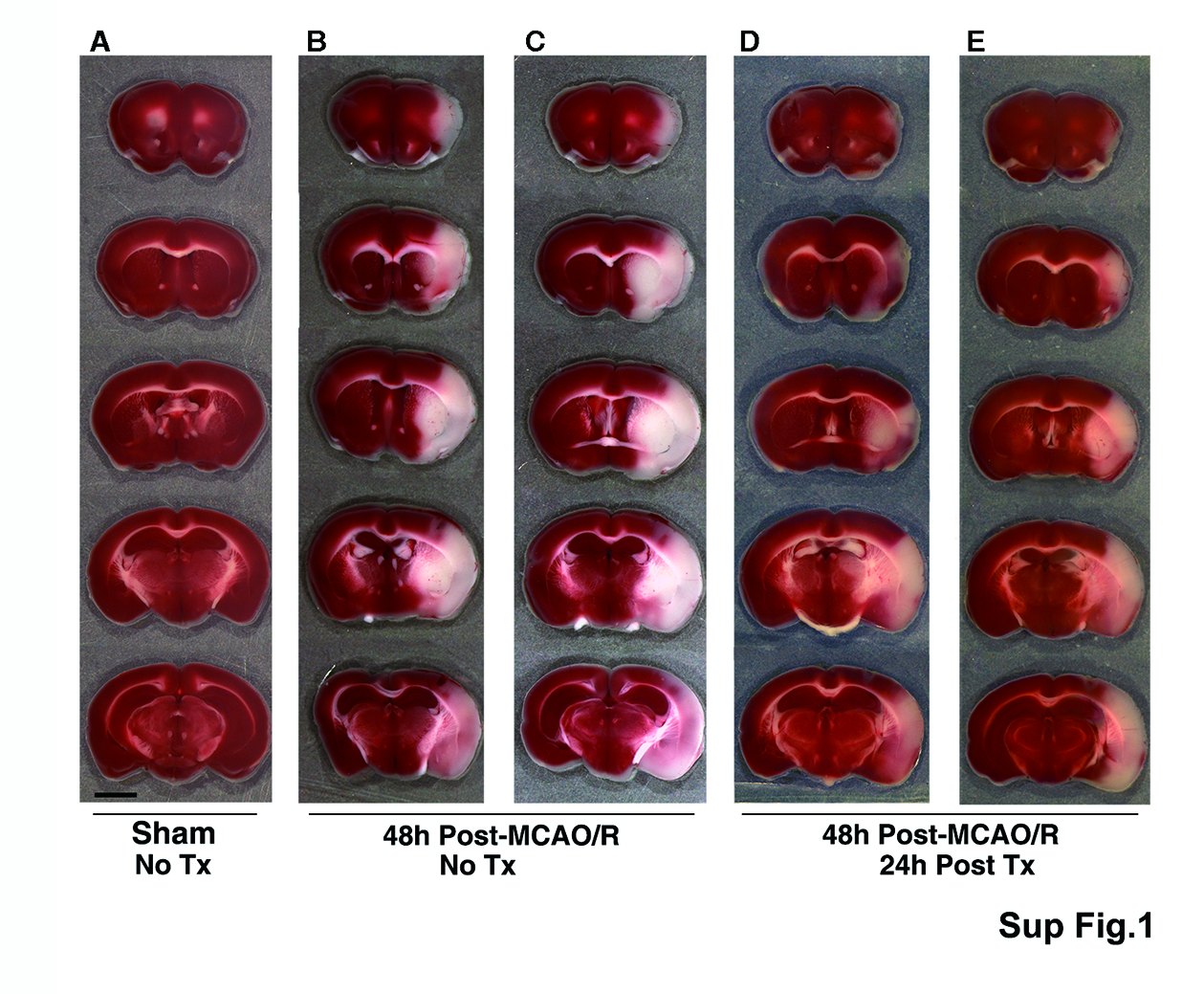

Supplement: Supplementary file 1 — Additional file 1: Figure S1: Human NSC transplantation reduces infarct volume. Infarct volume in MCAO/R mouse brains was measured using TTC staining (Red, viable tissue; White, infarct). Sixty minutes of MCAO and 48-hour reperfusion induced ischemic damage in both the caudo-putamen and cortex. Infarct volume was reduced in hNSC-transplanted mice (MCAO/R + Tx) 24 hours post-transplantation. Sections are from the same animal and are shown from anterior to posterior (top to bottom). A, anterior, P, posterior. Shown are (A) sham, (B, C) MCAO/R, and (D, E) MCAO/R with transplantation. Tx: transplantation, Scale bar, 3 mm. (TIF 3 MB) [file 13287_2014_439_MOESM1_ESM.tif]

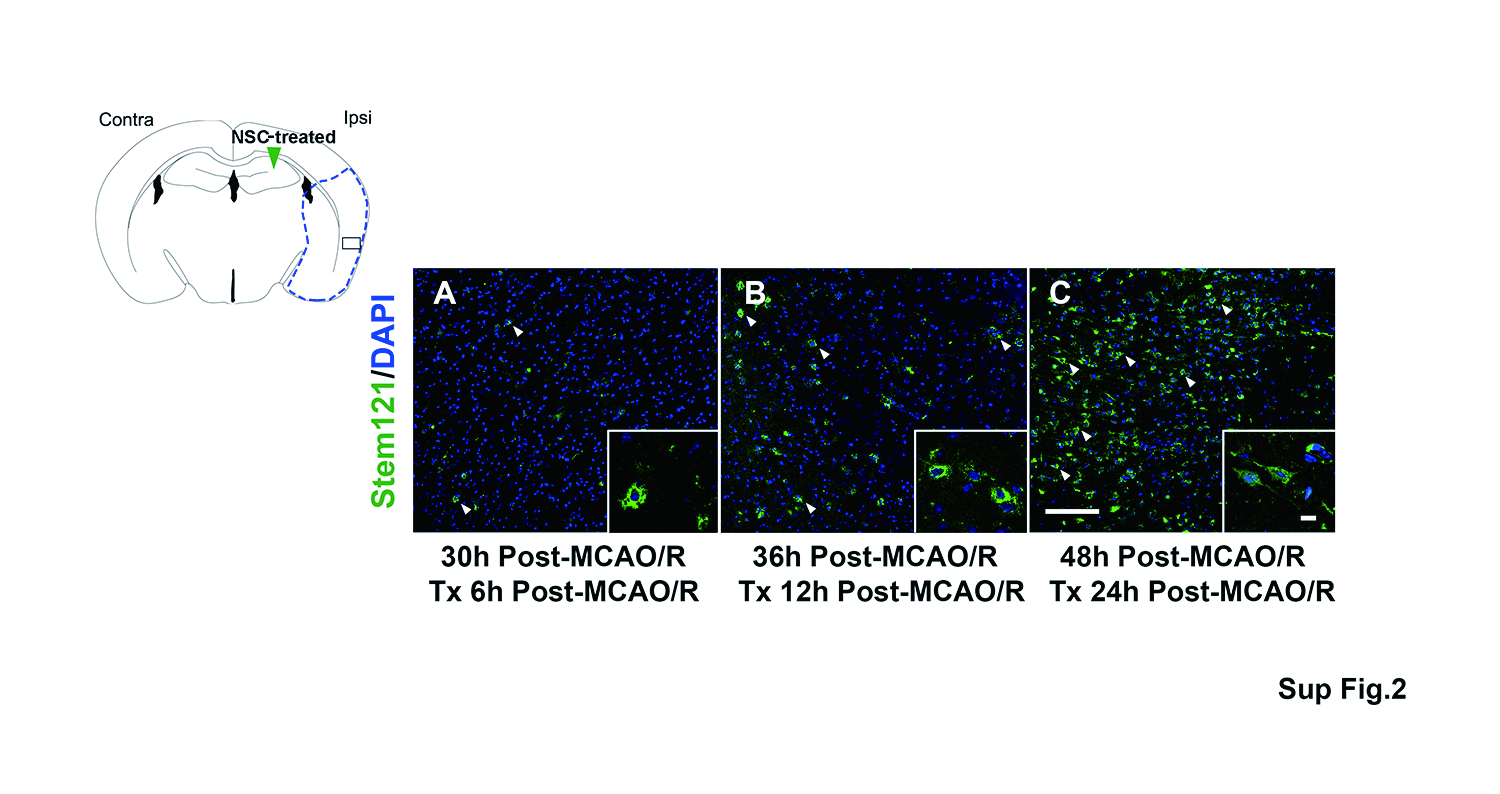

Supplement: Supplementary file 2 — Additional file 2: Figure S2: hNSC transplantation 24 hours post-MCAO/R shows the most extensive migration into stroke lesion. (A-C) Human NSCs were identified with the human cytoplasmic marker, Stem121 (green), following different transplantation time points: (A) 6 hours, (B) 12 hours, (C) 24 hours post-MCAO/R. (Insets, higher magnification images). White arrow indicates Stem121-positive cells. Sampling sites are shown in rectangle of diagram. Ipsi, ipsilesional; Contra, contralesional. Scale bar, 10 μm (100 μm inset). (TIFF 1 MB) [file 13287_2014_439_MOESM2_ESM.tiff]

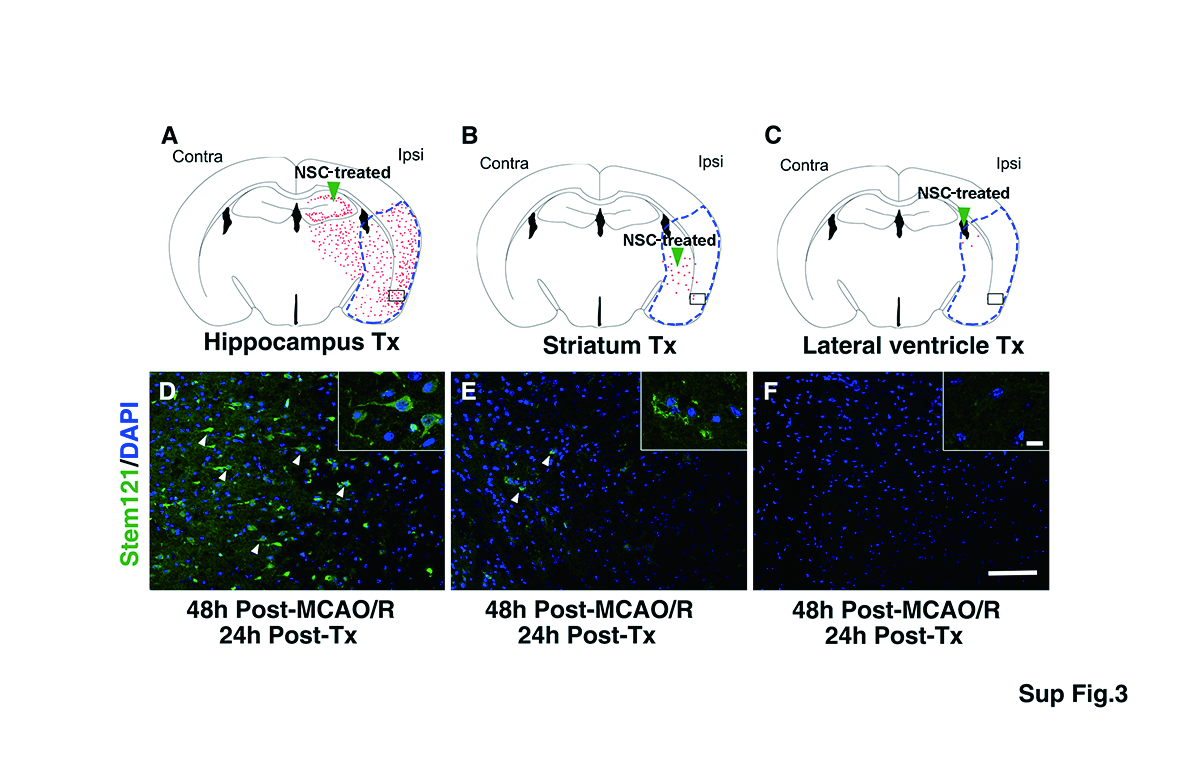

Supplement: Supplementary file 3 — Additional file 3: Figure S3: hNSCs transplanted into hippocampus 24 hours post-MCAO/R migrate most extensively into stroke lesion. (A-C) Diagrams show the distribution of NSCs 24 hours post-transplantation into the ipsilateral hippocampus, striatum, and lateral ventricle, respectively. Green arrows indicate transplantation site. Red dots indicate hNSC-disseminated areas. Ipsi, ipsilesional; Contra, contralesional. (D-F) hNSCs were identified with the human cytoplasmic marker, Stem121 (green). White arrows indicate Stem121-positive cells. The sampling sites are shown in the inset rectangles of A-C. Insets show higher magnification images. MCAO/R, middle cerebral artery occlusion with subsequent reperfusion. Scale bar, 10 μm (100 μm inset). Tx, transplantation. (TIF 1 MB) [file 13287_2014_439_MOESM3_ESM.tif]
